# A chemically modified antibody mediates complete eradication of tumours by selective disruption of tumour blood vessels

**DOI:** 10.1038/bjc.2011.78

**Published:** 2011-03-08

**Authors:** A Palumbo, F Hauler, P Dziunycz, K Schwager, A Soltermann, F Pretto, C Alonso, G F Hofbauer, R W Boyle, D Neri

**Affiliations:** 1 Department of Chemistry and Applied Biosciences, Institute of Pharmaceutical Sciences, Swiss Federal Institute of Technology Zürich, Wolfgang-Pauli-Strasse 10, Zürich 8093, Switzerland; 2 Department of Dermatology, University Hospital Zurich, Gloriastrasse 31, Zurich 8091, Switzerland; 3 Philochem AG, c/o ETH Zürich, Institute of Pharmaceutical Sciences, Wolfgang-Pauli-Strasse 10, HCI E520, Zürich 8093, Switzerland; 4 Institute of Surgical Pathology, University Hospital Zurich, Schmelzbergstrasse 12, Zurich 8091, Switzerland; 5 Department of Chemistry, University of Hull, Cottingham Road, Kingston-upon-Hull HU6 7RX, UK

**Keywords:** natural killer cells, photodynamic therapy, immunotherapy, monoclonal antibody, tumour neovasculature, squamous cell carcinoma

## Abstract

**Background:**

The possibility of eradicating cancer by selective destruction of tumour blood vessels may represent an attractive therapeutic avenue, but most pharmaceutical agents investigated so far did not achieve complete cures and are not completely specific. Antibody conjugates now allow us to evaluate the impact of selective vascular shutdown on tumour viability and to study mechanisms of action.

**Methods:**

We synthesised a novel porphyrin-based photosensitiser suitable for conjugation to antibodies and assessed anticancer properties of its conjugate with L19, a clinical-stage human monoclonal antibody specific to the alternatively spliced EDB domain of fibronectin, a marker of tumour angiogenesis.

**Results:**

Here we show in two mouse model of cancer (F9 and A431) that L19 is capable of highly selective *in vivo* localisation around tumour blood vessels and that its conjugate with a photosensitiser allows selective disruption of tumour vasculature upon irradiation, leading to complete and long-lasting cancer eradication. Furthermore, depletion experiments revealed that natural killer cells are essential for the induction of long-lasting complete responses.

**Conclusions:**

These results reinforce the concept that vascular shutdown can induce a curative avalanche of tumour cell death. Immuno-photodynamic therapy may be particularly indicated for squamous cell carcinoma of the skin, which we show to be strongly positive for markers of angiogenesis.

Aggressive solid tumours (Folkman, 2006) and haematological malignancies, such as lymphomas (Li *et al*, 2008; Sauer *et al*, 2009; Schliemann *et al*, 2009a, 2009b), myelomas (Atkins *et al*, 2008) and nests of leukaemia blasts in the bone marrow (Padro *et al*, 2000), depend on new blood vessels for their florid growth and dissemination. As angiogenesis is a rare event in the healthy adult (Carmeliet, 2003; Folkman, 2006), mainly confined to the female reproductive cycle, therapeutic strategies have been devised that aim at inhibiting the formation of new tumour blood vessels (inhibition of angiogenesis) or at destroying the pre-formed tumour neovasculature (vascular targeting).

Inhibition of key mediators of tumour angiogenesis has culminated in the clinical application of Avastin, a humanised monoclonal antibody binding soluble VEGF-A, thus stopping interaction with its receptors (Ferrara *et al*, 2004; Hurwitz *et al*, 2004). However, although Avastin confers a survival benefit to patients with colon, lung and kidney cancer when used in combination with chemotherapy (Hurwitz *et al*, 2004), this agent is rarely curative, as tumours often secrete multiple mediators of angiogenesis that compensate for the loss of VEGF-A activity.

In principle, strategies aimed at selective destruction of existing tumour blood vessels (rather than at inhibition of angiogenesis) could have a more profound impact on the disease, causing an avalanche of tumour cell death as a result of oxygen and nutrient deprivation (Neri and Bicknell, 2005). Indeed, the antibody-based targeted delivery of toxins or mild pro-coagulant factors to tumour blood vessels can lead to tumour infarction in mouse models of cancer. In some cases, complete tumour eradication was observed (Thorpe *et al*, 1985; Huang *et al*, 1997; Nilsson *et al*, 2001). Recently, tumour-targeting derivatives of a truncated form of tissue factor have been moved to mechanistic clinical trials in patients with cancer (Bieker *et al*, 2009).

Therapeutic strategies based on selective occlusion of tumour vasculature are marked with an inherent paradox. Although, it is clear that the collapse of tumour vasculature can lead to massive tumour cell death, it is less obvious that all tumour cells (including those at the periphery of the neoplastic lesion neighbouring on healthy and well-oxygenated tissues) should die as a result of a vascular insult. In fact, when solid tumours are treated with combretastatins (small organic molecules that interfere with tubulin polymerisation in endothelial cells causing a transient occlusion of tumour blood vessels (Chaplin *et al*, 2006)), extensive necrosis is observed only in the tumour core, whereas a rim of neoplastic cells survives at the periphery of the lesion and leads to progression of the disease (Chaplin *et al*, 2006; Heath and Bicknell, 2009).

In the study presented here, to investigate whether and how solid tumours can be completely eradicated solely as a result of a vascular shutdown, we experimentally disrupted tumour blood vessels by means of antibody–photosensitiser (PS) conjugates, which selectively localise to the tumour neovasculature *in vivo*. We had previously reported that new blood vessels induced in the cornea of rabbits could be completely ablated, following intravenous injection of suitable antibody–PS conjugates and irradiation with red light (Birchler *et al*, 1999). Moreover, we had observed that similar conjugates can trigger rapid intraluminal blood coagulation in small superficial tumours in mice (Fabbrini *et al*, 2006).

Photodynamic therapy (PDT) is a localised, non-invasive treatment modality for superficial malignancies that relies on the combination of non-toxic PSs and visible light in the presence of oxygen to generate cytotoxic reactive oxygen species. Photodynamic therapy causes localised cell death directly by induction of apoptosis and/or necrosis as well as indirectly by destruction of tumour vasculature and delayed stimulation of an immune response against the malignancy subjected to PDT (Castano *et al*, 2006).

Photosensitisers are small organic molecules (typically <1000 kDa) that absorb light and that may display a toxic action in their immediate surroundings either by generation of short-lived reactive oxygen species (such as singlet oxygen) (Josefsen and Boyle, 2008) or by localised heat generation (Chen *et al*, 1996). To facilitate immuno-PDT studies, we developed porphyrin-based PSs that absorb in the red light spectrum (approx. 630 nm) and that can be coupled to recombinant antibodies without compromising their immunoreactivity and solubility. As vascular tumour targeting agent, we used the human monoclonal antibody L19 specific to the alternatively spliced EDB domain of fibronectin, a marker of angiogenesis (Zardi *et al*, 1987). Extra domain B has identical sequence in mouse and man, which facilitates tumour targeting studies in syngeneic animal models (Tarli *et al*, 1999; Borsi *et al*, 2002; Berndorff *et al*, 2005). The small immune protein (SIP) format was chosen for the antibody, which exclusively localises to blood vessels within the tumour mass, as revealed by microautoradiographic studies (Borsi *et al*, 2002). Several biodistribution studies performed in tumour-bearing mice had previously indicated that the pharmacokinetic properties of antibodies in SIP format are intermediate between the ones of fast-clearing scFv fragments and those of IgG, and thus ideally suited for *in vivo* vascular tumour targeting applications (Borsi *et al*, 2002; Berndorff *et al*, 2005; Tijink *et al*, 2006). Indeed, SIP(L19) labelled with the radionuclide iodine-131 has recently shown its ability to preferentially localise to tumours in clinical trials and to induce complete responses in patients with radiosensitive lymphomas (Sauer *et al*, 2009). The L19 antibody has also been fused with many cytokines (Carnemolla *et al*, 2002; Halin *et al*, 2002, 2003; Gafner *et al*, 2006; Kaspar *et al*, 2007) and two of these derivatives (L19-TNF (Halin *et al*, 2003) and L19-IL2 (Carnemolla *et al*, 2002)) are currently being studied in various phase I and phase II clinical trials in patients with cancer.

The results presented in this study show that aggressive tumours, implanted in the skin of nude mice, can be completely eradicated as a result of vascular damage mediated by immuno-PDT using SIP(L19)–PS conjugates. Interestingly, complete tumour ablation required the presence of natural killer (NK) cells, as only partial inhibition of tumour growth could be observed following NK cell depletion with a monoclonal antibody specific to asialo-GM1.

These findings reinforce the concept that the selective ablation of tumour neovasculature can lead to long-lasting tumour eradication and may be of clinical significance for the treatment of squamous cell carcinoma (SCC).

Non-melanoma skin cancers such as basal cell carcinoma and SCC, as well as *in situ* forms of SCC, represent the most frequent type of cancer in the fair-skinned population. Their incidence is increasing world wide, with immunocompromised patients being particularly affected (Hofbauer *et al*, 2010). At present, SCC and related malignancies are either removed by excision or treated with non-surgical options such as radiotherapy and PDT.

## Materials And Methods

### Chemistry

The PS used for conjugation to SIP(L19) was 5-[4-(succinimide-*N*-oxycarbonyl)phenyl]-10,15,20-tris-(4-*N*-methylpyridimiumyl)porphyrin trichloride and was synthesised from commercially available materials and is described in detail in the Supplementary Online Materials.

### Animals and cell lines

WI-38 VA-13 fibroblasts (ATCC number CCL-75.1), F9 murine teratocarcinoma cells (ATCC number CRL-1720) and A431 human epidermoid carcinoma cells (ATCC number CRL-1555) were maintained in exponential growth in DMEM medium adjusted to contain 4 mM L-glutamine, 4.5 g l^−1^ glucose, 10% foetal bovine serum, 100 U ml^−1^ penicillin and 100 *μ*g ml^−1^ streptomycin (Gibco-Invitrogen, Basel, Switzerland). Six- to eight-week-old female Balb/c Nude CAnN.Cg-Foxn1nu/Crl mice were obtained from Charles River Laboratories (Sulzfeld, Germany). All animal experiments were performed under a project license granted by the Veterinäramt des Kantons Zürich (169/2008).

### Antibodies

The cloning, expression and purification of SIP(L19), SIP(F8) and SIP(F16) antibodies have been described previously (Borsi *et al*, 2002; Brack *et al*, 2006; Zuberbuhler *et al*, 2009). Briefly, SIP cDNA was cloned into the pCDNA 3.1 vector (Invitrogen, Basel, Switzerland). The construct was transfected in CHO-S cells, and selected stable high-expressing clones were grown in PowerCHO-Cd 2 medium (Lonza, Belgium) and used to produce the antibodies, which were purified directly from the supernatant by protein A affinity chromatography.

### Preparation of photoimmunoconjugates

Small immune protein–PS (SIP–PS) conjugates were prepared as follows: ∼20-fold molar excess of amine-reactive porphyrin (PS 10 mg ml^−1^ in DMSO) was added to purified SIP antibody (1 mg ml^−1^, in PBS pH 7.4) and incubated for 4 h at 30°C, gently shaking, avoiding light exposure. Small immune protein–PS conjugates were then purified from free reagent over a PD-10 column (GE Healthcare, Glattburg, Switzerland) and dialyzed overnight against PBS pH 7.4 at 4°C.

Small immune protein–PS conjugates were analysed by SDS–PAGE under reducing and non-reducing conditions using the Invitrogen PAGE system following the manufacturer's instructions. The gels were first imaged under a Cy5 filter lamp and then stained with Coomassie brilliant blue. The labelling ratio was estimated spectroscopically by measuring the absorbance at 280 nm for the SIP (assuming that a 1 mg ml^−1^ of SIP solution gives absorption of 1.4 U at 280 nm) and at 422 nm for the PS (*ε*=159 100 M^−1^ cm^−1^). In addition, mass spectrometric analysis was used to assess the labelling ratio. For MALDI-TOF/TOF MS analysis, the conjugates were mixed with a sinapinic matrix (10 mg ml^−1^ in 50% CAN and 0.05% TFA) at a 1:4 dilution and spotted onto a MALDI target plate. Analysis of samples was performed on a freshly calibrated AB4800 MALDI-TOF/TOF mass spectrometer (Applied Biosystems, Carlsbad, CA, USA). For data analysis, the Data Explorer software (version 4.8) of Applied Biosystems was used. A Superdex 200 size-exclusion column (GE Healthcare) was used to analyse the gel filtration profile of the antibodies before and after PS conjugation under native conditions using fast protein liquid chromatography (GE Healthcare).

### Photokilling assay

For the *in vitro* photocytotoxicity assay WI-38 VA-13 fibroblasts were used by seeding 30 000 cells per well in a 96-well plate and incubating overnight at 37°C in 5% CO_2_. The next day medium was removed and cells were incubated with 50 *μ*l of SIP or SIP–porphyrin conjugate in the appropriate dilutions (in PBS) for 1 h at 37°C. Cells were subsequently washed with PBS twice to remove unbound antibodies, and cells were covered with 50 *μ*l of PBS. The cells were then irradiated using a KL 1500 electronic tungsten halogen lamp (Zeiss, Jena, Germany) equipped with a 620/60 filter (Chroma, Bellows Falls, VT, USA) for a total light dose of 60 J cm^−2^. After light treatment, PBS was removed and 100 *μ*l of fresh medium were added. The cells were then incubated at 37°C, 5% CO_2_ atmosphere over night. Controls include SIP–porphyrin conjugate without irradiation, PBS only with irradiation, unmodified SIP with irradiation and medium only without irradiation. The following day, cell viability was measured using the Cell Titer 96Aqueous One Solution Cell Proliferation Assay (Promega, Dübendorf, Germany), following the manufacturer's instructions. The percentage of cell growth was calculated as a ratio of the counts between treated cells over the relative control (cells treated with SIP–porphyrin conjugate without light exposure).

### Mouse model and PDT protocols

(A) F9 murine teratocarcinoma cells (3 × 10^6^) were injected subcutaneously into the flank of Balb/c nude mice. When tumours were established and clearly palpable (∼50 mm^3^), mice were randomly distributed among the groups (4 animals per group) and irradiated with a laser (Ceralas PDT diode laser 635±3 nm cw 2 W, light dose of 60 J cm^−2^) 24 and 48 h after intravenous injection in the tail lateral vein of 150 *μ*g of SIP(L19)–PS. In addition to SIP(L19)–PS, control groups included mice receiving SIP(L19)–PS in the absence of light, mice treated with an irrelevant antibody–PS conjugate (SIP(F16)-PS) and mice treated with saline. For depletion of NK cells (Habu *et al*, 1981), mice were treated with 0.3 mg of anti-asialo GM1 antibody (Wako, Osaka, Japan) by intraperitoneal injection every 5 days. Mice were anesthetised with ketamine (40 mg kg^−1^)/xylazine (6 mg kg^−1^) before light irradiation. Differences between different groups were compared using Student's *t*-test. (B) A431cells (3 × 10^6^) were injected subcutaneously into the flank of Balb/c nude mice. When tumours were established and clearly palpable (50–100 mm^3^), mice were irradiated once with a laser (Ceralas PDT diode laser 635±3 nm cw 2 W, total light dose of 60 J cm^−2^) 24 h after a single intravenous injection in the tail lateral vein of 150 *μ*g of SIP(L19)–PS conjugate. Mice were monitored daily and tumour growth was measured three times per week with a digital caliper using the following formula: volume=length × width × width × 0.5. Mice were killed when the tumour reached a volume >2000 mm^3^. In addition, mice were photographed using a digital reflex camera (Nikon D90).

Detailed procedures used for the analysis of tissues (HC, IHC/IHF, infiltration study) are reported in the Supplementary Online Materials.

## Results

### Synthesis of conjugatable PS derivatives

5-(4-Carboxyphenyl)-10,15,20-tri-(4-pyridyl)porphyrin was used as starting material to introduce an activated ester that allows the conjugation of this PS to amino residues on proteins.

**Figure 1:**
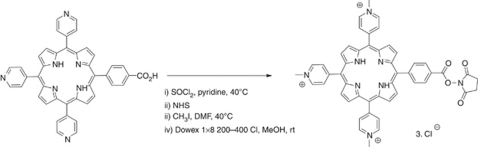
Synthetic route to the photosensitiser 5-[4-(succinimide-*N*-oxycarbonyl)phenyl]-10,15,20-tris-(4-*N*-methylpyridimiumyl)porphyrin trichloride.

To transform the carboxylic acid into the *N*-hydroxysuccinimide ester, it was first converted to the corresponding acyl chloride by reaction with thionyl chloride (Figure 1). The resulting acid chloride was then reacted with *N-*hydroxysuccinimide. The final step of the reaction sequence involved quaternisation with methyl iodide to give the tricationic porphyrin. Iodide counter-ions were exchanged for chlorides to increase water solubility as described previously (Sutton *et al*, 2002).

### Preparation and *in vitro* characterisation of antibody–PS conjugates

**Figure 2:**
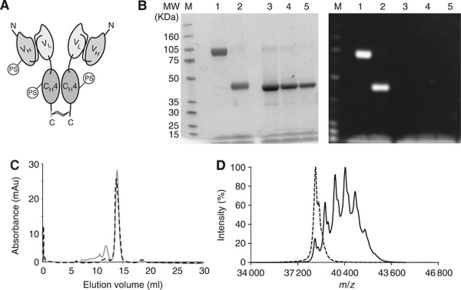
Antibody–photosensitiser (PS) conjugates. (**A**) Schematic representation of an antibody in SIP format, consisting of a scFv fragment fused to an *ε*-C_H_4 domain of human IgE, which mediates non-covalent homodimerisation of the recombinant antibody molecule. A C-terminal cysteine residue mediates the formation of covalent homodimers between the two subunits. Some of the primary amino groups of the antibody are covalently modified with the PS described in Figure 1. (**B**) SDS–PAGE analysis (left panel, Coomassie blue staining; right panel, Cy5 fluorescence imaging) of SIP(L19)–PS: lanes 1 and 2, SIP(L19)–PS in non-reducing and reducing conditions, respectively; lanes 3–5, SIP(L19) standards of equal volume (0.8, 0.4 and 0.2 mg ml^−1^, respectively) in reducing conditions. (**C**) Size-exclusion chromatography profile of SIP(L19) (dashed line) and of SIP(L19)–PS conjugate (solid grey line). (**D**) MALDI TOF/TOF mass spectra for SIP(L19) (dashed line) and SIP(L19)–PS conjugate (solid line).

Figure 2A presents the schematic structure of an antibody in SIP format, where certain lysine residues have been covalently modified with an amine-reactive PS moiety. Figure 2B shows the results of SDS–PAGE analysis of SIP(L19) covalently modified with PS, using Coomassie blue staining and fluorescence detection methods. Complete formation of a disulphide-linked covalent homodimer can be observed, which is disrupted when the sample is run in reducing conditions. No free PS is detectable in the sample. Figures 2C and D present size-exclusion chromatography and mass spectrometric analysis of SIP(L19) before and after conjugation with PS, indicating that the majority of the antibody conjugate elutes with the retention expected for a covalent homodimer, while exhibiting a Poisson distribution of molecular masses in the fine MS analysis, reflecting a statistical labelling of primary amino groups. At the average stoichiometric ratio of monomeric SIP(L19)/PS=1:3 (Figure 2D), the conjugate exhibited >90% retention of immunoreactivity, as revealed by affinity chromatography on antigen resin.

**Figure 3:**
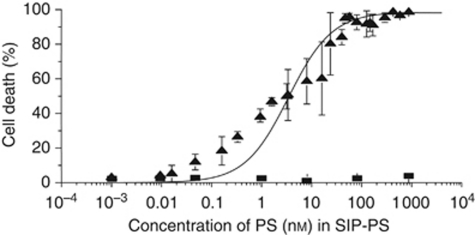
Photocytotoxicity of SIP(L19)–PS (▴) and SIP(L19) (▪), measured on WI-38 VA-13 fibroblasts using the MTS assay (obtained values are the mean of at least three independent experiments±s.d.). The curve is a fit of the data using the Hill equation.

Small immune protein(L19)–PS is a non-internalising antibody conjugate. In line with previous observations (Birchler *et al*, 1999; Fabbrini *et al*, 2006), this conjugate is able to kill cells when it accumulates in their proximity given the presence of red light. Figure 3 presents the results of a photokilling experiment performed with WI38VA-transformed fibroblasts, which secrete EDB-containing fibronectin (Carnemolla *et al*, 1996). When irradiated with 150 J cm^−2^ of red light, the target cells could be completely killed in the presence of 100 nM SIP(L19)–PS conjugate (LD_50_=3 nM), whereas no detectable cell killing was observed without irradiation.

### *In vivo* characterisation of antibody–PS conjugates

**Figure 4:**
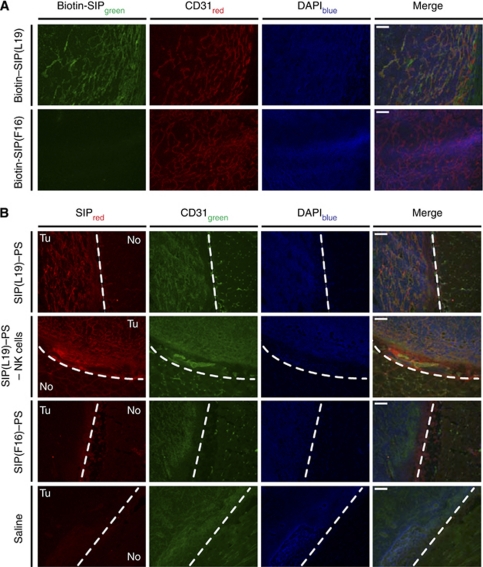
*In vivo* localisation of conjugates: *ex vivo* immunofluorescence analysis. (**A**) Binding of SIP(L19) and SIP(F16) to F9 tumour tissue evaluated by fluorescent microscopic analysis of tumour sections after incubation with the biotinylated SIPs. (**B**) Fluorescent microscopic analysis of F9 tumour sections to assess tumour targeting by SIP(L19)–PS (without or with previous depletion of NK cells), SIP(F16)–PS or saline 24 h after injection. Dashed white line, border between tumour (Tu) and normal (No) tissue. CD31, marker for endothelial cells. Scale bars, 100 *μ*m.

Small immune protein(L19) exclusively stains blood vessels in frozen sections of F9 tumours, as revealed by immunofluorescence analysis. By contrast, SIP(F16) does not recognise any antigen in the mouse (Brack *et al*, 2006), did not stain mouse F9 tumours in our experiment (Figure 4A) and therefore was chosen as negative control for therapy experiments. To confirm that SIP(L19)–PS retains the vascular tumour targeting properties of the unmodified antibody, we injected nude mice bearing F9 tumours with this conjugate and detected its *in vivo* localisation by immunofluorescence analysis of tissue sections from animals killed 24 h after intravenous injection of the conjugate. Exclusive localisation to tumours could be observed, with high selectivity for the tumour neovasculature (Figure 4B).

### Complete eradication of tumours by selective disruption of tumour blood vessels

One molecule of homodimeric SIP(L19) was coupled to an average of six PS molecules (Figure 2), resulting in a dose of PS administered to the mice equal to 0.6 mg kg^−1^. This is an order of magnitude less than doses typically required to cure tumours in mice using PDT based on similar PSs (Korbelik and Dougherty, 1999).

**Figure 5:**
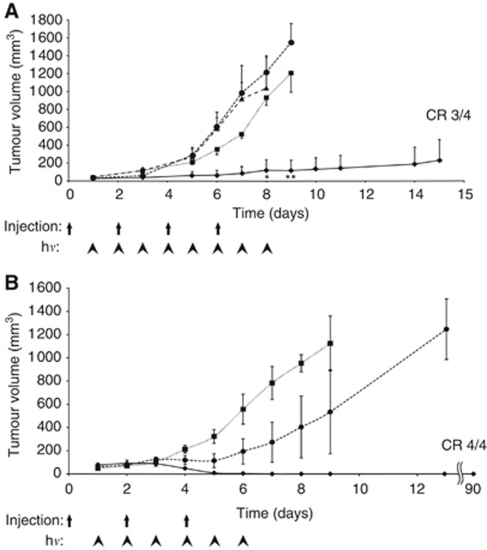
Therapeutic activity of SIP(L19)–PS. (**A**) Nude mice bearing subcutaneous F9 teratocarcinomas were injected intravenously with 150 *μ*g of SIP(L19)–PS (⧫ and •), SIP(F16)–PS (▴) or saline (▪) on days 0, 2, 4 and 6 of the treatment schedule, and tumours were irradiated daily with 60 J cm^−2^ from days 1 to 8 (⧫, ▴ and ▪) or not irradiated at all (•); ^*^P<0.01 *vs* not irradiated, ^**^P<0.01 *vs* saline. (**B**) Nude mice bearing subcutaneous F9 teratocarcinomas were injected with 150 *μ*g of SIP(L19)–PS (⧫ and •) or saline (▪) on days 0, 2 and 4, and tumours were irradiated daily with 60 J cm^−2^ from days 1 to 6. Mice in one of the groups receiving PS conjugate were depleted of NK cells by injections of 0.3 mg of an anti-asialo-GM1 antibody every 5 days during therapy, starting at day −1 of the therapy regimen (•). CR, complete response. h*ν*, irradiation.

**Figure 6:**
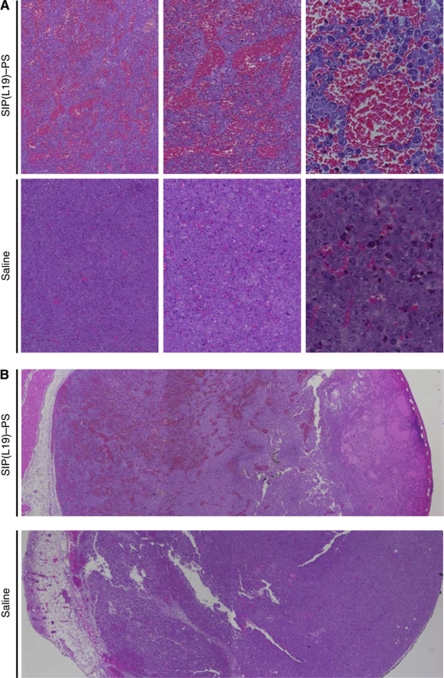
Effect of PDT with SIP(L19) –PS on tumour histology. Sections of F9 tumours excised 1 h after irradiation, stained with haematoxylin/eosin, photographed at × 2.5 (**B**), × 5 (**A**, left panel), × 10 (**A**, middle panel) and × 40 (**A**, right panel) magnification.

To evaluate the *in vivo* therapeutic activity of SIP(L19)–PS, we injected this conjugate in tumour-bearing mice (150 *μ*g per mouse) and irradiated tumours with red light (two doses of 60 J cm^−2^). Mice treated with SIP(L19)–PS exhibited a strong anticancer response, with three out of four complete eradications that were long lasting (Supplementary Figure S1 and Figure 5A). In contrast, mice receiving saline or SIP(F16)–PS displayed rapid tumour growth despite irradiation. Similarly, mice treated with SIP(L19)–PS in the absence of light did not exhibit any inhibition of tumour growth (Figure 5A). The therapeutic action of SIP(L19)–PS was due to the rapid photodynamic disruption of tumour blood vessels, leading to extensive haemorrhage and oedema throughout the tumour mass (Figure 6), as well as to widespread pyknosis and karyorrhexis presaging massive tumour cell death.

**Figure 7:**
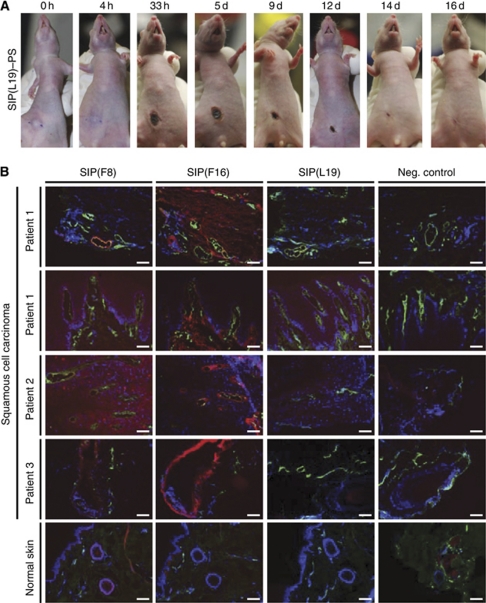
Immunophotodynamic application for skin lesions. (**A**) Subcutaneous xenografts of human A431 epidermoid carcinoma in nude mice are ablated and heal upon a single dose of immuno-PDT with SIP(L19)–PS. Tumour-bearing nude mice injected with SIP(L19)–PS conjugate, photographed at several points in time after a single dose of irradiation with light. (**B**) Sections of skin tissue (SCC or normal skin) from human patients stained for nuclei (DAPI, blue), relevant target antigens (detected with SIPs F8, F16 and L19, respectively; red) and vascular endothelial cells (outlined by anti-von Willebrand factor antibody, green). Scale bars, 100 *μ*m.

We further tested SIP(L19)–PS in mice bearing A431 tumours as a model of SCC of the skin. To mimic a clinical procedure for the treatment of SCC patients, we injected the antibody–PS conjugate in mice bearing subcutaneous A431 tumours in a single dose (150 *μ*g), followed by 3 min irradiation (60 J cm^−2^) 24 h later. Although tumours in saline-injected mice were not affected by irradiation, A431 lesions in mice receiving SIP(L19)–PS were rapidly converted into black scabs (Figure 7A) as a result of selective disruption of the tumour vasculature. The lesions healed completely and with excellent cosmetic outcome within 2 weeks of their immuno-photodynamic ablation (Figure 7A) and did not grow back for the subsequent 100 days after treatment.

### Essential role of NK cells in complete tumour eradication

The complete tumour eradication observed as a result of selective vascular damage is counter-intuitive, as one would expect tumour cells at the periphery (i.e., adjacent to normal, well-perfused tissue) to survive and re-grow (Chaplin *et al*, 2006; Heath and Bicknell, 2009). To investigate whether NK cells could contribute to tumour eradication following vascular disruption, immuno-PDT with SIP(L19)–PS was performed in the presence or absence of NK cell depletion (Figure 5B). As in the previous experiment, complete tumour eradication (four out of four mice) was observed in animals treated with SIP(L19)–PS. By contrast, tumours continued to grow in mice that had received saline treatment. Interestingly, removal of NK cells (mediated by an anti-asialo GM1 antibody) before and during immuno-photodynamic treatment with SIP(L19)–PS abrogated the therapeutic effect, resulting only in transient tumour growth retardation followed by progression (Figure 5B).

**Figure 8:**
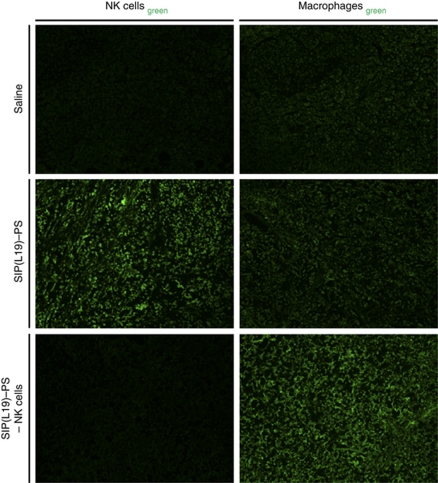
Lymphocyte infiltration of tumours following PDT with SIP(L19)–PS. Microscopic immunofluorescence analysis of F9 tumour sections revealing the degree of infiltration by NK cells and macrophages, respectively, 6 h after PDT based on saline or SIP(L19)–PS without or with previous depletion of NK cells. Magnification: × 10.

Natural killer cells efficiently infiltrated tumours within 6 h after immuno-PDT treatment (Figure 8). In contrast, tumours of mice injected with saline or depleted of NK cells before therapy were virtually devoid of NK cells. The levels of macrophages were similar in tumours of mice treated with SIP(L19)–PS or saline. However, infiltration of tumours by macrophages increased substantially in immuno-PDT experiments where NK cells were depleted before therapy (Figure 8), possibly attempting to compensate for the missing population of NK cells.

### Immunofluorescence analysis of human specimens of SCC

To investigate whether immuno-PDT strategies based on vascular targeting antibodies could be used for the ablation of skin lesions of SCC, we first performed an analysis by immunofluorescence samples of human SCCs and normal skin, using in addition to L19, the F8 and F16 human antibodies, specific to the alternatively spliced EDA and the A1 domain of tenascin-C, respectively (Schliemann *et al*, 2009b). Like the EDB, these extra-domains are virtually undetectable in normal adult tissues (Neri and Bicknell, 2005), but are often abundantly expressed around tumour blood vessels in primary tumours and in metastases (Neri and Bicknell, 2005). Figure 7B shows the immunofluorescence analysis of SCC lesions obtained from a small cohort of patients, in which a selective staining of vascular and stromal structures could be observed for the L19, F8 and F16 antibodies. Furthermore, these antibodies are able to strongly stain frozen sections of an SCC-like mouse tumour based on the A431 epidermoid carcinoma cells, xenografted subcutaneously in nude mice (Supplementary Figure S2).

## Discussion

In this article, we have shown that an antibody–PS conjugate specific to the alternatively spliced EDB domain of fibronectin, a marker of angiogenesis, has the ability to localise selectively around tumour blood vessels *in vivo*. This allows selectively damaging the tumour vasculature by irradiation with red light, leading to massive tumour cell death and to lasting cures. Depletion experiments indicated that the action of NK cells is essential for the induction of complete cancer eradication, killing cells neighbouring on normal tissue at the periphery of the tumour.

In the past, selective damaging of tumour neovasculature has been studied with antibody–toxin and antibody–tissue factor conjugates (Thorpe *et al*, 1985; Ran *et al*, 1998), as well as with pharmacological agents (e.g., combretastatins (Hinnen and Eskens, 2007), ASA404 (Head and Jameson, 2010)) capable of preferential interaction with tumour endothelial cells (Chaplin *et al*, 2006; Martinelli *et al*, 2007; Siemann *et al*, 2009). Indeed, this latter class of vasculature-disrupting agents has been investigated in clinical trials in patients with cancer, revealing that combretastatin-A4 can lead to enhanced tumour responses when combined with radiotherapy or anticancer drugs (i.e., carboplatin, paclitaxel) (Siemann *et al*, 2009) and that the flavonoid ASA-404 potentiates the action of taxane-based chemotherapeutic regimens in patients with non-small-cell lung cancer (Head and Jameson, 2010).

In principle, vasculature-disrupting agents like combretastatins, while having a clear impact on vascular structures of tumours (Heath and Bicknell, 2009), could act in part also on other cells of tumours and stroma (Heath and Bicknell, 2009). Furthermore, when used as single agents, combretastatins induce only a transient shutdown of tumour blood vessels and do not cure cancer (Heath and Bicknell, 2009). By contrast, the use of vascular targeting antibody derivatives (Neri and Bicknell, 2005) allows to confirm experimentally the selective localisation of the therapeutic agent on the tumour neovasculature by a variety of experimental techniques, including *ex vivo* fluorescence microscopy (Figure 4B) or microautoradiographic analysis (Borsi *et al*, 2002; Villa *et al*, 2008). The use of PSs as bioactive payloads ensures that therapy starts precisely when tumours are irradiated, that is, at a point in time when the concentration of antibody conjugate on non-vascular tumour cells and in normal tissue is negligibly low. Indeed, anti-EDB antibodies localise to the subendothelial extracellular matrix around tumour blood vessels (Borsi *et al*, 2002; Niesner *et al*, 2002), and antibody-delivered PSs mediate the disruption of adjacent tumour vasculature by acting on proximal endothelial cells through diffusible reactive oxygen species (Josefsen and Boyle, 2008) or through local generation of heat (Chen *et al*, 1996; Yu *et al*, 2010).

At present, approved clinical applications of PDT are mainly limited to localised skin cancers (such as basal cell carcinoma and *in situ* SCC), actinic keratosis, head and neck carcinomas and the premalignant condition Barrett's oesophagus. Light penetration of tissues reaches a maximum of only several millimetres at wavelengths around 750–800 nm (Wan *et al*, 1981), thus limiting practical applications to superficial cancer or endoscopically accessible lesions. However, the additional selectivity associated with the antibody-based delivery of PSs promises to extend the applicability of this methodology, while limiting side effects.

Our results show that human SCC of the skin can be selectively targeted by SIPs and that human skin tumours implanted in nude mice can be ablated in a curative manner by one single dose of immuno-PDT based on a vascular targeting antibody–PS conjugate. Complete responses were achieved at a dramatically reduced dose of PS compared with conventional (non-targeted) PDT regimens. These findings suggest that the therapeutic efficacy of PDT in the clinical treatment of human skin cancers may be greatly enhanced by the selective delivery of PSs conjugated to vascular tumour-targeting antibodies. At the same time, we envisage a significant reduction of curative PS doses and of side effects in such targeted immuno-PDT regimens.

Clinical trials are now needed to elucidate whether the promising results obtained with SIP(L19)–PS in the A431 tumour model are confirmed in patients with cutaneous SCC or other EDB-positive skin lesions. If so, then antibody–PS conjugates such as SIP(L19)–PS may greatly extend the applicability of immuno-PDT in the clinic, sparing many skin cancer patients the more invasive treatment modalities of surgery and radiotherapy.

Independently of any practical application of immuno-PDT (Kuimova *et al*, 2007), the data presented in this study unequivocally confirm that a selective damage of the tumour neovasculature leads to an avalanche of tumour cell deaths and is sufficient for complete and long-lasting eradication of tumours that are not cured by conventional chemotherapy (Ebbinghaus *et al*, 2005).

Natural killer cells play a crucial role in the mechanism of action of anticancer therapeutic antibodies (e.g., rituximab, trastuzumab), engaging the Fc portion of the antibody with Fc-γ receptor 3a (FCGR3A, CD16), leading to degranulation (Cartron *et al*, 2002). High-affinity variants in the FCGR3A polymorphism have been associated with better response to therapeutic antibodies (Cartron *et al*, 2002). Furthermore, protein engineering strategies that increase the binding affinity of antibodies to the cognate Fc receptor may dramatically potentiate the anticancer activity of therapeutic antibodies (Umana *et al*, 1999; Presta *et al*, 2002; Nimmerjahn and Ravetch, 2005). The activity of NK cells can be boosted by the targeted delivery of certain pro-inflammatory cytokines by means of vascular targeting antibodies (Carnemolla *et al*, 2002; Halin *et al*, 2002, 2003; Borsi *et al*, 2003; Schliemann *et al*, 2009a). Our discovery of the important role played by NK cells in the eradication of cancer following selective disruption of tumour blood vessels may provide a strong rationale for the combination of vascular disrupting agents with immunostimulatory drugs (e.g., immunocytokines) in cancer therapy.
