# Supplementary material for: A chemically modified antibody mediates complete eradication of tumours by selective disruption of tumour blood vessels
Source: Br J Cancer. 2011 Mar 8;104(7):1106–15. doi: 10.1038/bjc.2011.78 (PMC3068510; doi:10.1038/bjc.2011.78)
Supplement: Supplementary Materials [file bjc201178x1.doc]

**SUPPLEMENTARY MATERIALS**

**A chemically-modified antibody mediates complete eradication of tumors by selective disruption of tumor blood vessels.**

**Authors:** Alessandro Palumbo^1^, Felix Hauler^1^, Piotr Dziunycz^2^, Kathrin Schwager^1,3^, Alex Soltermann^4^, Francesca Pretto^1^, Cristina Alonso^5^, Günther Hofbauer ^2^, Ross W. Boyle^5^, and Dario Neri^1^.

**Affiliations:** ^1^Institute of Pharmaceutical Sciences, Department of Chemistry and Applied Biosciences, Swiss Federal Institute of Technology Zürich, Wolfgang-Pauli-Strasse 10, CH-8093 Zürich, Switzerland; ^2^Department of Dermatology, University Hospital Zurich, Gloriastrasse 31 CH-8091 Zurich, Switzerland; ^3^Philochem AG, c/o ETH Zürich, Institute of Pharmaceutical Sciences, Wolfgang-Pauli-Strasse 10, HCI E520, CH-8093 Zürich, Switzerland; ^4^Institute of Surgical Pathology, University Hospital Zurich, Schmelzbergstrasse 12, CH-8091 Zurich, Switzerland; ^5^Department of Chemistry, University of Hull, Cottingham Road, HU6 7RX, Kingston-upon-Hull, United Kingdom.

**Corresponding Authors:**

Dario Neri, neri@pharma.ethz.ch **and** Ross W. Boyle, r.w.boyle@hull.ac.uk.

**Supplementary Figure**

`
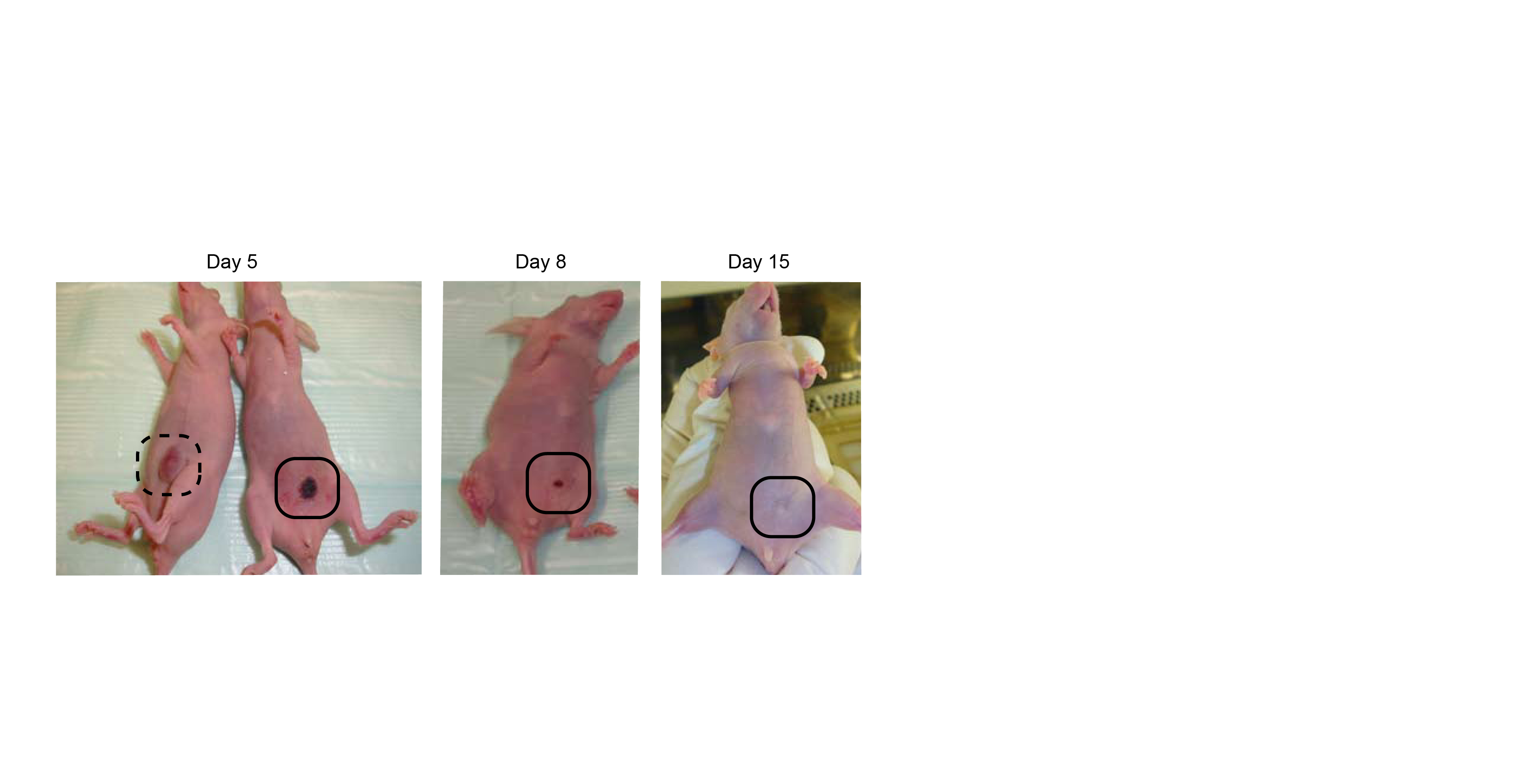


**Figure S1: Macroscopic illustration of therapeutic activity of SIP(L19)-PS.** Left panel, tumor-bearing mice receiving injections of PBS (dashed line) and SIP(L19)-PS (solid line) during photodynamic therapy, photographed on day 5 of the treatment schedule (see **Figure 5**); middle panel, mouse treated with SIP(L19)-PS, photographed on day 8; right panel, mouse treated with SIP(L19)-PS, photographed on day 15.

**
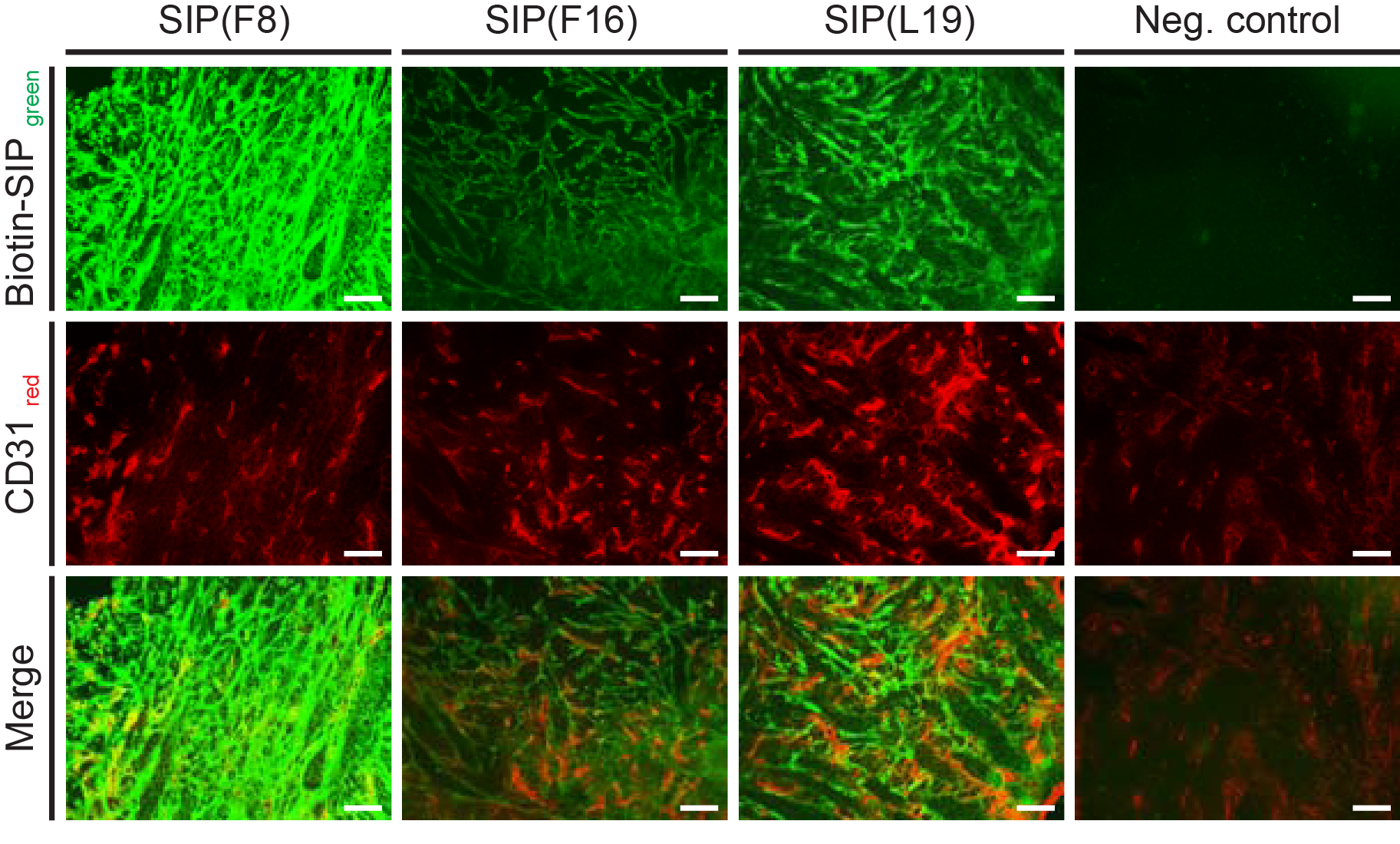
**

**Figure S2: Immunofluorescence analysis of subcutaneous tumors.** Relevant target antigens (SIPs, green) and vascular endothelial cells (outlined by anti mouse CD31 antibody, red) detected on human A431 tumour sections from nude mice. Scale bars, 100 µm.

**Supplementary Materials and Methods**

**Chemistry**

The photosensitizer (PS) used for conjugation to SIP(L19) was 5-[4-(succinimide-*N*-oxycarbonyl)phenyl]-10,15,20-tris-(4-*N*-methylpyridimiumyl)porphyrin trichloride and was synthesized from commercially available materials as described below:

### 5-(4-carboxyphenyl)-10,15,20-tri-(4-pyridyl)porphyrin

To a refluxing mixture of 4-formylbenzoic acid (2.54 g, 17 mmol) and 4-pyridinecarboxaldehyde (9.78 mL, 104 mmol) in acetic acid (200 mL) and nitrobenzene (150 mL) was added dropwise pyrrole (4 mL, 57.8 mmol). The reaction mixture was kept under reflux and intense stirring for 1 h. After this period the solvents were removed under reduced pressure and the residue was purified by flash chromatography using a mixture of 15% dichloromethane / methanol as eluent. 5-(4-carboxyphenyl)-10,15,20-tri-(4-pyridyl)porphyrin was obtained pure, after precipitation from methanol / chloroform, in 6% yield (510 mg).

### 5-[4-(succinimide-*N*-oxycarbonyl)phenyl]-10,15,20-tri-(4-pyridyl)porphyrin

To a stirred solution of 5-(4-carboxyphenyl)-10,15,20-tri-(4-pyridyl)porphyrin (51.1 mg, 0.077 mmol) in dry pyridine (5 mL) was slowly added thionyl chloride (0.1 mL, 1.37 mmol, 18 equiv.). The reaction was then stirred at 50º C, protected from light and atmospheric moisture, for 30 min. After this period *N-*hydroxysuccinimide (200 mg, 1.74 mmol, 22.6 equiv.) was added and the mixture maintained under the previous conditions for 3 h. The pyridine was then removed under vacuum, the residue taken up in dichloromethane and washed with aqueous saturated sodium carbonate. The organic layer was dried over anhydrous sodium sulphate and evaporated to dryness under reduced pressure. Crystallization of the residue from light petroleum / chloroform afforded pure 5-[4-(succinimide-*N*-oxycarbonyl)phenyl]-10,15,20-tri-(4-pyridyl)porphyrin in 90% yield (52.3 mg).^1^H NMR (CDCl_3_, 400 MHz), *δ*: -2.90 (br s, 2H, NH), 3.03 (br s, 4H, CH_2_), 8.17 (dd, 6H, 10,15,20-H-Ar-*o* , *J* 1.6, 4.3 Hz), 8.37 (dd, 2H, 5-H-Ar-*m*, *J* 1.7, 6.5 Hz), 8.57 (dd, 2H, 5-H-Ar-*o*, *J* 1.7, 6.5 Hz), 8.85 (d, 2H, H-β, *J* 4.8 Hz), 8.86-8.90 (m, 6H, H-β), 9.06 (dd, 6H, 10,15,20-H-Ar-*m*, *J* 1.6, 4.3 Hz).

### 5-[4-(succinimide-*N*-oxycarbonyl)phenyl]-10,15,20-tris-(4-*N*-methylpyridimiumyl)porphyrin triiodide

To a stirred solution of 5-[4-(succinimide-*N*-oxycarbonyl)phenyl]-10,15,20-tri-(4-pyridyl)porphyrin (26 mg, 0.0343 mmol) in dry DMF (5 mL) was added a large excess of methyl iodide (0.5 mL, 8.03 mmol) via syringe. The reaction mixture was kept under stirring at 40º C, under nitrogen atmosphere and protected from light overnight. The product was then precipitated with diethyl ether in order to remove the DMF and any trace of methyl iodide. The resulting solid was filtered, dissolved in acetone / water (50:50) and the solvents removed under reduced pressure. After a second precipitation from acetone / water, pure 5-[4-(succinimide-*N*-oxycarbonyl)phenyl]-10,15,20-tris-(4-*N*-methylpyridimiumyl)porphyrin triiodide was obtained in 89% (31.6 mg) yield. ^1^H NMR (CDCl_3_, 400 MHz), *δ*: -3.05 (br s, 2H, NH), 3.01 (br s, 4H, CH_2_), 4.71 and 4.72 (2s, 6+3H, CH_3_), 8.50 (d, 2H, 5-H-Ar-*m*, *J* 8.3 Hz), 8.60 (d, 2H, 5-H-Ar-*o*, *J* 8.3 Hz), 9.00 (d, 6H, 10,15,20-H-Ar-*o*, *J* 6.6 Hz), 9.04-9.13 and 9.14-9.22 (2m, 8H, H-β), 9.48 (d, 6H, 10,15,20-H-Ar-*m*, *J* 6.6 Hz).

### 5-[4-(succinimide-*N*-oxycarbonyl)phenyl]-10,15,20-tris-(4-*N*-methylpyridimiumyl)porphyrin trichloride

To a solution of 5-[4-(succinimide-*N*-oxycarbonyl)phenyl]-10,15,20-tris-(4-*N*-methylpyridimiumyl)porphyrin triiodide (56.9 mg, 0.0481 mmol) in anhydrous methanol (57 mL) was added Dowex 1x8 200-400 Cl (1.81 g). The reaction was protected from moisture and light and allowed to stir at room temperature for 1 h. The resin was removed by filtration and the reaction mixture concentrated under reduced pressure. Pure 5-[4-(succinimide-*N*-oxycarbonyl)phenyl]-10,15,20-tris-(4-*N*-methylpyridimiumyl)porphyrin trichloride was obtained as a brown solid after precipitation with acetone (41.8 mg, 96%). ^1^H NMR (DMSO-d6, 400 MHz), *δ*:-3.05 (br s, 2H, NH), 3.01 (br s, 4H, CH_2_), 4.72 and 4.73 (2s, 6+3H, CH_3_), 8.50 (d, 2H, 5-H-Ar-*m*, *J* 8.0 Hz), 8.60 (d, 2H, 5-H-Ar-*o*, *J* 8.0 Hz), 9.00 (d, 6H, 10,15,20-H-Ar-*o*, *J* 5.6 Hz), 9.05-9.12 and 9.13-9.24 (2m, 8H, H-β), 9.49 (d, 6H, 10,15,20-H-Ar-*m*, *J* 5.6 Hz). ^13^C NMR (DMSO-d6, 100 MHz), *δ*: 25.7 (*C*H_2_), 47.9 (*C*H_3_), 115.0, 115.5, 120.7, 124.6, 128.9, 132.1 (10,15,20-Ar-*o*-C), 135.1, 144.2 (10,15,20-Ar-*m*-C), 147.3, 156.4, 162.0 (CO_2_N), 170.5 (CON).

**Immunofluorescence with biotinylated SIPs**

10 µm cryostat sections of murine F9 teratocarcinomas were fixed in ice-cold acetone, rehydrated in PBS pH 7.4 and blocked with 20% donkey/goat serum in PBS (Invitrogen, Switzerland). Sections were then incubated with rat anti-mouse CD31 antibody (BD Pharmigen) and with either biotinylated SIP(L19) or biotinylated SIP(F16). Detection was performed using Alexa Fluor 594-labelled donkey anti-rat IgG (Invitrogen, Switzerland) and streptavidin conjugated to Alexa Fluor 488 (Invitrogen, Switzerland). Nuclei were stained with DAPI (Invitrogen, Switzerland). Slides were mounted with Glycergel mounting medium (Dako, Denmark) and images were captured on an Axioskop 2 mot plus microscope equipped with an AxioCam MRc camera (Zeiss, Germany).

***Ex vivo* analysis of targeting**

150 µg of SIP(L19)-PS, SIP(F16)-PS or saline were injected into the lateral tail vein of balb/c nude mice bearing subcutaneously injected F9 teratocarcinomas. Animals were sacrificed 24 h after injection of conjugate, tumor and organs were excised, embedded in cryoembedding medium (Microm, Germany) and stored at -80º C. 10 µm cryostat sections were cut and fixed in ice-cold acetone, rehydrated in PBS pH 7.4 and blocked with 20% donkey/goat serum in PBS. SIP-PS was detected with rabbit anti-human IgE antibody (Dako, Denmark) followed by Alexa Fluor 594-labelled goat anti-rabbit IgG (Invitrogen, Switzerland). Rat anti-mouse CD31 antibody was applied to outline endothelial cells using Alexa 488-labelled donkey anti-rat IgG as secondary antibody (Invitrogen, Switzerland). Slides were mounted with glycergel mounting medium and images were captured on a Axioskop 2 mot plus microscope equipped with an AxioCam MRc camera.

**Analysis of the effects of photodynamic treatment on tumor histology**

Balb/c nude mice bearing subcutaneously injected F9 teratocarcinomas (average size of 200 - 250 mm^3^) were injected with 150 µg of SIP(L19)-PS or saline and irradiated once after 24 h, with a total light dose of 60 J/cm^2^. 1 h after treatment, mice were sacrificed, tumors were excised, fixed in 4% paraformaldehyde and embedded in paraffin. Tumor sections of 4 µm were stained with hematoxylin/eosin. Tumors were scored for presence of necrosis, hemorrhage and edema as well as thrombosis of blood vessels.

**Immunofluorescence analysis of tumor infiltration by lymphocytes**

For immunofluorescence analysis of lymphocytic infiltration following therapy, Balb/c nude mice bearing subcutaneously injected F9 teratocarcinomas (average size of 200 – 250 mm^3^) were injected with 150 µg of SIP(L19)-PS or saline and irradiated after 24 h, with a total light dose of 60 J/cm^2^. For NK cell depletion, mice were treated with 0.3 mg of Anti asialo GM1 antibody (Wako, Japan) by *i.p.* injection 2 days before the experiment. Tumors were excised 6 h after irradiation, embedded in cryoembedding medium (Microm, Germany) and stored at -80º C. 10 µm cryostat sections were cut and fixed in ice-cold acetone, rehydrated in PBS pH 7.4 and blocked with 20% donkey/goat serum in PBS (Invitrogen, Switzerland). Then, sections were incubated with the primary antibodies rat anti-mouse F4/80 (anti-macrophage; Abcam) or rabbit anti-asialo-GM1 (anti-NK; Wako Pure Chemical Industries) in 12% bovine serum albumin in PBS for 1 h at room temperature or overnight at 4° C. Sections were washed 3 × 5 min with PBS at room temperature and then incubated with fluorescent Alexa 488-coupled secondary antibodies (BD Biosciences Pharmigen) and DAPI (Invitrogen, Switzerland) in 12% bovine serum albumin / PBS. Finally, sections were washed 3 × 5 min in PBS and mounted with Glycergel (Dako) and a cover glass (VWR International). Images were obtained using the individual fluorescent channels on an Axioskop 2 mot *plus* (Carl Zeiss).

***Immunofluorescence analysis of tumors with biotinylated SIPs***

10 μm cryostat sections of human skin tissue samples (normal skin or squamous cell carcinoma, provided by the Department of Dermatology, University Hospital Zurich, Switzerland) and sections of human A431 carcinomas xenografted into nude mice were fixed in ice-cold acetone, rehydrated in PBS pH 7.4 and blocked with 20% donkey/goat serum in PBS (Invitrogen, Switzerland). Sections were then incubated with rabbit anti-human von Willebrand factor antibody (Dako, Denmark) or rat anti-mouse CD31 antibody (BD Pharmigen), respectively, to outline vascular endothelial cells and with either biotinylated SIP(F8), SIP(F16) or SIP(L19). Detection was performed using Alexa Fluor 488-labeled goat anti-rabbit IgG or Alexa Fluor 594-labeled donkey anti-rat IgG (Invitrogen, Switzerland), respectively, and streptavidin conjugated to Alexa Fluor 594 or 488 (Invitrogen, Switzerland), respectively. Nuclei were stained with DAPI (Invitrogen, Switzerland). Slides were mounted with fluorescent mounting medium (Dako, Denmark) and images were captured on an Axioskop 2 mot plus microscope equipped with an AxioCam MRc camera (Zeiss, Germany).
